# Therapeutic Notes

**Published:** 1890-09

**Authors:** 


### THERAPEUTIC NOTES.

Hypodermic Injections of Ergot in Facial Neuralgia. — For the relief of facial neuralgia hypodermic injections of ergot are incomparably superior to aconite or gelsemium. Any one who has used it will never resort to either of the above named remedies. I have used it the last six years, and have never had it fail in but one case. In that case there was evidently organic disease. Ordinarily one injection relieves the pain permanently; sometimes two; and in one very severe and obstinate case which had gone through the hands of several physicians without relief, it required three. After the third injection he never had a twinge of pain. I put it in the temple, as nearly over the seat of pain as convenient. I used the plain extract, and have it made on purpose for hypodermic use. One minim represents two grains of ergot. Of this I use from eight to twelve minims blood-warm, at one injection, and without diluting. In order to make this a success, two things are essential: one is, to have a fresh and pure article of ergot to make the extract from; and the other is, to have the extract reasonably fresh. If kept long, it is not only worthless, but irritating. When properly prepared and fresh, it produces more or less pain for ten or fifteen minutes, and when the pain from the injection subsides the neuralgia is usually gone, and does not return.

I have used this treatment for sciatica and other forms of neuralgia, but not with very satisfactory results. — Dr. Stewart in the *Peoria Med. Monthly*.

---

Bad Breath.— Dr. Frank H. Gardner, in the *Dental Review*, speaks of the causes of bad breath. He concludes: First, decaying particles in the mouth as far back as the pharynx vault taint the breath, if exhaled, very little, if at all. Second, mouth-breathers have a bad breath when the tonsils are enlarged, or when cheesy masses exist in the tonsillary mucous folds. Third, certain gastric derangements taint the breath only when gases are eructated through the mouth. Fourth, the principal cause of bad breath is decomposition in the intestinal canal, the retention of fecal matter in the transverse and descending colon, and the absorption of gases into the circulation, finally exhaled by the lungs. Fifth, catarrh, nasal, pharyngeal or bronchial, causes bad breath. Sixth, medicines or aliments which undergo chemical changes below the esophagus may, by rapid absorption through the stomach walls, or immediately below, give to the breath the characteristic odor. Bad breath is often a source of serious annoyance to patients, and the fact that it has more than a local cause is too often ignored by the physician, who therefore fails to cure it.

---

Effects of Close Shaving.— A writer in the *Medical Classics* looked through a microscope at a closely shaved face, and he reports that the skin resembled a piece of raw beef. "To make the skin perfectly smooth requires," he says, "not only the removal of hair, but also a portion of the cuticle, and a close shave means the removal of a layer of skin all around. The blood-vessels thus exposed are not visible to the eye, but under the microscope each little quivering mouth holding a minute blood-drop protests against such treatment. The nerve tips are also uncovered and the pores are left unprotected, which makes the skin tender and unhealthy. This sudden exposure of the inner layer of the skin renders a person liable to have colds, hoarseness, and sore throat.

---

Poke Berries a Satisfactory Anti-Fat.— Several years ago, I called attention to the efficacy of pills made from the extract of poke berries as a reliable remedy in obesity. My attention was attracted to it from the fact that birds that feed on poke berries in the Fall are deficient in adipose tissues. It has been my custom for several years to gather, in the Fall after frost, a quantity of the berries, express their juice, and evaporate it to the consistency of an extract, of which I make pills of three or four grains. The dose is two pills before each meal, sometimes increased to three or four. They diminish the appetite to some extent. In some cases the reduction of weight is remarkable, as much as fifteen to twenty pounds per month.— M. M. Griffith, M. D., in *American Hom.*

---

Of the treatment of summer diarrhea, Waugh, of Philadelphia, says:

This is the third Summer in which I have been using the sulpho-carbolate of zinc as a remedy in summer complaint. As yet I have no reason to alter the opinion I formed two years ago: that this is the safest and most effectual remedy at our command for producing intestinal asepsis. In simple diarrhea, the alkaline syrup of rhubarb, with the addition of a little tincture of hydrastis (a drachm to the ounce of syrup), answers every purpose; but if this does not promptly cure, or if the stools continue to be offensive, or if the case looks at all serious, I order at once the sulpho-carbolate, in doses of one-half to two grains every two hours, in powder, with a little bismuth.

The Removal of Moles.—Moles on the face are now being successfully treated by the use of sodium ethylate. The mole is painted with the sodium ethylate, a fine glass rod being used. When the mole has a varnished look, the ethylate is gently rubbed in with the glass rod to make it penetrate more deeply. The mole turns nearly black, and a hard crust forms over it, which is nearly three weeks in becoming detached. When it comes off, the mole is much lighter than before, and this treatment can be continued until the mark is scarcely noticeable.

---

Treatment of Incontinence of Urine in Children.— It is very important (*Annales d' Orthopedie*) to recognize the cause of the incontinence. If local causes have to be excluded, the probable cause is a simple nervous debility ; puberty will probably end this trouble. Local causes should be treated, if diagnosed. If the urinary passages are too irritable, belladonna will give good results ; if the sphincter lacks tonicity, strychnine will doubtless prove useful. It is impossible to predict what medicament should be used.—*Times and Register*.

---

Ice in the Night Sweats of Phthisis.— Rosenbach (*Pr. Med. Wochensch.*) recommends for arresting the night sweats, which so enfeeble the phthisical, applications over the abdomen of a bladder half full of ice, and to let it remain several hours. The patients tolerate it very well, even when they present an evening elevation of temperature. This means succeeds where atropine has failed, as well as powdered salicylate of soda sprinkled over the entire body.—*N. Y. Med. Times*.

---

A Test for the Purity of Drinking Water is given as follows by Prof. Angell of the Michigan University : Dissolve about half a teaspoonful of the purest white sugar in a pint bottle, completely full of water to be tested, tightly stopped ; expose it to daylight and a temperature up to seventy degrees Fahr. After a day or two examine, holding the bottle against something black, for floating specks, which will betray the presence of organic matter in considerable proportion.—*Practice*.

---

Pancoast holds that as men are made drunk sooner when standing or sitting while taking alcohol, than when in a recumbent posture, in like manner it takes less ether to produ ce anesthesia if the patient sits up.

FOR ECZEMA OF DENTITION, treatment is to be directed to three indications (*Gazette Hebdom.* in *Annals of Gynecology and Pediatry*, July, 1890.)

1. *To calm pruritus of the gums, frequent rubbing with the finger dipped in a solution of the following:*

| | |
|---|---|
| ℞—Cocaine hydrochlorat | gr. j |
| Potass. bromid | gr. x. |
| Glycerin | |
| Aqua destillat | āā f℥ss. M. |

2. *For insomnia a dessertspoonful hourly of—*

| | |
|---|---|
| ℞—Sodii bromidi | gr. xij |
| Syrup aurant. flor | f℥iij M. |

3. *For the local eczema the following:*

| | |
|---|---|
| ℞—Zinci oxid | gr. xx. |
| Vaselini | ℥j. M. |

---

POORLY nourished persons who suffer from constipation should have a generous diet. Those who can do so without discomfort should drink cream. The next best dietetic remedy is fresh butter, which they should use freely on their foods. Cocoa to a certain degree is nourishing, and, therefore, as a beverage is preferable to coffee or tea, provided, of course, it is well borne.—*Boston Journal of Health.*

---

ONE of the very best applications for the bites of mosquitoes and fleas, also for other eruptions attended with intense itchings, is menthol in alcohol, one part to ten. This is very cooling and immediately effectual. It is also an excellent lotion for application to the forehead and temples in headache, often at once subduing the same.—*Boston Journal of Health.*

---

EARACHE.—Take five drachms of camphorated chloral, thirty parts of glycerine, and ten parts of the oil of sweet almonds. A piece of cotton is saturated and introduced well into the ear; and it is also rubbed behind the ear. The pain is relieved as if by magic, and, if there is inflammation, it often subsides quickly.—*Times and Register.*

---

HOW TO DETECT THE MORPHINE HABIT.—An efficient means of detecting the morphine habit is by adding a few drops of perchloride of iron to the patient's urine. A characteristic blue tinge results if he is a morphine user.—*N. Y. Med. Times.*

